# Human Immunodeficiency Virus (HIV) Genetic Diversity Informs Stage of HIV-1 Infection Among Patients Receiving Antiretroviral Therapy in Botswana

**DOI:** 10.1093/infdis/jiab293

**Published:** 2021-06-02

**Authors:** Manon Ragonnet-Cronin, Tanya Golubchik, Sikhulile Moyo, Christophe Fraser, Max Essex, Vlad Novitsky, Erik Volz

**Affiliations:** 1 MRC Centre for Global Infectious Diseases Analysis, Imperial College London, London, United Kingdom; 2 Big Data Institute, University of Oxford, Oxford, United Kingdom; 3 Botswana Harvard AIDS Initiative, Gaborone, Botswana; 4 Department of Immunology and Infectious Diseases, Harvard T.H. Chan School of Public Health, Boston, Massachusetts, USA; 5 Brown University, Providence, Rhode Island, USA

**Keywords:** ART, early HIV infection, HIV, HIV treatment, NGS

## Abstract

**Background:**

Human immunodeficiency virus (HIV)-1 genetic diversity increases during infection and can help infer the time elapsed since infection. However, the effect of antiretroviral treatment (ART) on the inference remains unknown.

**Methods:**

Participants with estimated duration of HIV-1 infection based on repeated testing were sourced from cohorts in Botswana (n = 1944). Full-length HIV genome sequencing was performed from proviral deoxyribonucleic acid. We optimized a machine learning model to classify infections as < or >1 year based on viral genetic diversity, demographic, and clinical data.

**Results:**

The best predictive model included variables for genetic diversity of HIV-1 *gag*, *pol*, and *env*, viral load, age, sex, and ART status. Most participants were on ART. Balanced accuracy was 90.6% (95% confidence interval, 86.7%–94.1%). We tested the algorithm among newly diagnosed participants with or without documented negative HIV tests. Among those without records, those who self-reported a negative HIV test within <1 year were more frequently classified as recent than those who reported a test >1 year previously. There was no difference in classification between those self-reporting a negative HIV test <1 year, whether or not they had a record.

**Conclusions:**

These results indicate that recency of HIV-1 infection can be inferred from viral sequence diversity even among patients on suppressive ART.

## Teaser

A single HIV virus is usually transmitted. HIV then replicates, making errors, and over time genetic diversity increases. We found that time since HIV infection can be estimated from within-patient HIV genetic diversity, even when patients are on treatment.

Accurate inference of human immunodeficiency virus (HIV)-1 infection stage is crucial for estimating HIV incidence and to evaluate the population-level effectiveness of antiretrovirals and other interventions. Identifying recent HIV infections is also critical to estimating their contribution to onward transmission [1–6]. The Fiebig staging system classifies early HIV infection based on a combination of diagnostic assay results, including tests for viral ribonucleic acid (RNA) and the p24 viral antigen [7]. Then, in the first few months of infection, time since seroconversion can be estimated based on serological assays, which measure the type and strength of immune responses. After infection, HIV-specific antibodies increase, and antibody test cutoffs can distinguish between recent and chronic infections [8, 9]. However, the window period for detecting recent infections using serological assays is limited to approximately 4 months, after which antibody levels reach a plateau [8, 9]. Furthermore, many factors influence the performance of serological assays, including country of origin, race/ethnicity, disease progression [10], and, importantly, HIV-1 subtype [9]. Thus, there is a rationale for developing complementary methods for identifying recent infections.

Sequencing data can be used to estimate HIV genetic diversity within hosts, and so genetic sequences may provide an alternative biomarker to inform stage of HIV infection [11–13]. Most HIV infections are established by a single founder virus, and viral diversity within a host increases over time [14]. Therefore, the number of ambiguous nucleotide bases produced by population-based sequencing can be used to distinguish recent from chronic infections [11, 12]. Next-generation sequencing (NGS) enables precise identification of viral haplotypes and calculation of viral population diversity within hosts. Pairwise diversity estimates derived from NGS thus yield a more accurate estimation of time since infection [13, 15]. Accumulation of genetic diversity also indicates time since infection with the hepatitis C virus [16].

Most published studies seeking to identify recent infections have been conducted on samples from recent diagnoses and known to be antiretroviral therapy (ART) naive. However, in population-based cohorts, thousands of individuals have been sequenced without knowledge of infection timing or treatment initiation [17]. For example, the PANGEA Consortium has sequenced HIV from over 18 000 individuals across sub-Saharan Africa. In Botswana, at one of the PANGEA sites, initiation of treatment at diagnosis (universal ART) was rolled out from 2016 onwards, and over 6000 individuals have been sequenced through PANGEA. Classifying those infections as recent or chronic is important for downstream analysis of incidence trends and transmission patterns. Because many PANGEA participants were on fully suppressive ART, it was not always possible to generate HIV sequences from viral RNA in plasma; instead, viral sequences were generated from proviral deoxyribonucleic acid (DNA). An additional question is whether changes in viral diversity are maintained among treated patients within proviral DNA sequences to the extent that diversity-based metrics for identifying recent infections can still be applied.

We determined whether HIV infections could be classified as being more recent or older than 1 year based on NGS sequence diversity metrics, among a cohort of participants in Botswana, the majority of whom were on ART and many sequenced from proviral DNA.

## METHODS

### Data

Participant data were obtained from 3 different cohorts that included participants with duration of infection known to be less or more than 1 year and for whom full genome NGS sequences were available. Next-generation sequencing was performed by the BioPolymers Facility at Harvard Medical School (https://genome.med.harvard.edu/) and through collaboration with the PANGEA HIV consortium [17, 18] (www.pangea-hiv.org) using Illumina platforms MiSeq and HiSeq, as previously described [19–21]. Assembly and alignment methods for these samples have been detailed elsewhere [22]. Sequences were subtyped using REGA [23]. We used sequences from a single time point for each participant. Samples were collected across 3 studies: BHP012 [24], Mochudi [25], and the Botswana Combination Prevention Project (BCPP) [25]. The BHP012 study ran from 2004 to 2008 and screened participants for HIV infection by a combination of enzyme immunoassay and HIV-1 RNA testing to recruit recently infected patients based on the estimated date of seroconversion [24]. Participants from the Mochudi study were tested for HIV-1 antibodies annually from 2010 to 2013, and seroconverters were identified based on a negative then a positive test [25]. Most data originated from BCPP, a community-randomized trial conducted from 2013 to 2018 across 30 villages in Botswana [26]. We classified BCPP infections as recent if participants had a documented negative HIV test less than 1 year before their positive diagnosis at the beginning of the trial or whether participants seroconverted during the trial with a documented negative test less than 1 year prior. The BCPP infections were classified as chronic if participants were HIV positive at enrollment and had documented evidence of a positive HIV test >1 year before the trial. Demographic and clinical data were available for most participants, including age, sex, viral load, sample date, and ART status. Because sample dates were so strongly associated with cohort of sampling, we did not include them as a predictor in our models. Human immunodeficiency virus sequences and associated epidemiological and clinical data utilized within the study are available upon request to the PANGEA consortium (https://www.pangea-hiv.org/).

### Calculating Genetic Diversity

We calculated the genetic diversity at each site in the HIV genome using 2 statistics: Entropy, denoted *H*, and the mean pairwise difference, denoted π. These are defined as follows:


$$\begin{array}{l} H=-\underset{k=1}{\overset{4}{\sum }}\;\;x\log x \end{array}$$

and


$$\begin{array}{l} \pi =1-\underset{k=1}{\overset{4}{\sum }}\;\;x^{2} \end{array}$$

Where 𝑘 takes the value of each nucleotide in turn (A, C, T G) and 𝑥 takes the relative frequency of each nucleotide in turn. For each gene (*gag*, *pol*, and *env*), we then calculated average entropy and π, eliminating sites with coverage <100 after deduplication. Entropy and π were log-transformed for analysis.

### Logistic Regression and Machine Learning (xgboost) Models

All analyses were performed in R 3.6.1, using the packages caret [27], pROC [28], and xgboost [29]. We split our data repeatedly into training (70%) and testing (30%) datasets to evaluate a series of logistic regression models. Predictors included the following: log entropy and/or log π for each gene (*gag*, *pol*, *env*), gender, age, log viral load, and ART status. We ran models with and without interactions between diversity and ART status and interactions between diversity and viral load. We then evaluated the ability of each model to predict the probability of being recent (0–1) for each sample, by calculating sensitivity, specificity, and balanced accuracy for a range of thresholds. Models were optimized for balanced accuracy (which optimizes the sum of sensitivity and specificity to improve identification across both classes), and we assessed the robustness of estimates through cross-validation (1000 replicates).

Next, we fitted the xgboost machine learning algorithm, again predicting probability of recency and including diversity metrics and/or demographic and clinical predictors. We compared performance (as measured by balanced accuracy) of the xgboost models through cross-validation (1000 replicates).

### Reliability of Self-Reported Human Immunodeficiency Virus Testing History

Our classifier was then evaluated on a separate dataset. At enrollment, BCPP participants were asked when they had last been tested for HIV (if at all), what the test result was, and whether they had a record of that result. Using our best-fit prediction algorithm, we predicted recency for 3 groups of participants: (A) those with recorded evidence of a negative test within the last year (note that these individuals were removed from the training dataset for this iteration of the model), (B) those who self-reported a negative HIV test within the last year but had no record, and (C) those who self-reported a negative HIV test more than 1 year ago but had no record. We then compared the frequencies of predicted recent and chronic infections between groups A and B and groups B and C using Fisher’s exact test. Because the xgboost model generates for each sample the probability of recency rather than a binary prediction, we also compared the probability distributions between both pairs of groups using the Kolmogorov-Smirnov (KS) test.

## RESULTS

### Genetic Diversity Is Affected by Stage of Infection and Antiretroviral Treatment Status

Stage of infection could be classified as < or >1 year for 1944 participants: 209 recent (20% on ART) and 1735 chronic (93% on ART) participants. Most participants originated from the BCPP trial [26], supplemented by seroconverters from BHP012 (n = 39) [8] and Mochudi (n = 16) [9]. Most sequences were subtype C (1875 of 1943, 96.5%), remnant sequences were subtypes A1, B, F1, and C recombinants. There was a marked difference in age between participants with recent versus chronic infections (Table 1).

**Table 1.** Demographic and Clinical Characteristics of Individuals With Known Recent and Chronic Infections^a^

Variable	Category	Recent	Chronic
Total		209	1735
Study	BCPP	154	1735
	BHP012	39	0
	Mochudi	16	0
ART status	Treated	41	1621
	Untreated	168	99
	NA	0	15
Age	Mean (±SD)	29.71 (**±**10.33)	42.78 (**±**10.09)
Sex	F	162	1322
	M	47	413
Viral load, log_10_ copies/mL	Mean (±SD)	3.58 (**±**1.27)	1.86 (±0.78)
	NA	6	0

Abbreviations: ART, antiretroviral treatment; BCPP, Botswana Combination Prevention Project; NA, not applicable; SD, standard deviation.

^a^Viral loads were log-transformed before calculating the mean for each group. Undetectable viral loads, which indicate viral suppression, are recorded as 40 copies/mL, because that is the lower limit of the viral load assay used.

**Figure 1.**
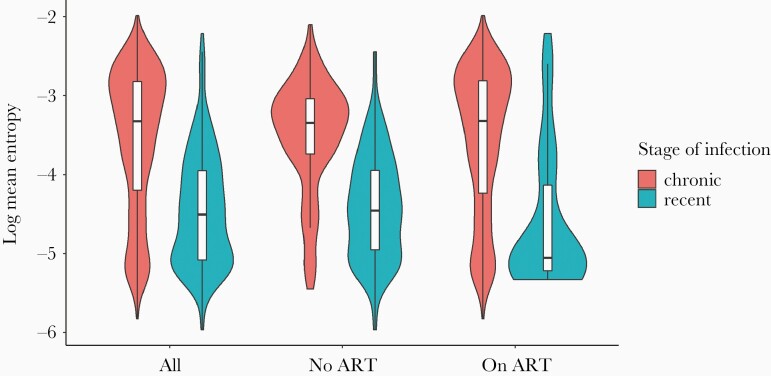
Viaplot of log mean entropy for participants based on stage of infection (chronic and recent) and antiretroviral treatment (ART) status (naive or treated). Log mean entropy for recent infections (−4.45; −5.33 to −2.70) was significantly below that of chronic infections (−3.57; −5.34 to −2.34). Averaged across *gag*, *pol*, and *env*.

There was a statistically significant difference in genetic diversity between recent and chronic infections, as estimated through entropy or π (KS test D = 0.47, *P* < 10–16) (Figure 1). Nonetheless, there was considerable overlap in diversity distributions, particularly among individuals on ART (Figure 1). In addition, the range of diversity among recent infections was substantial, reflecting the divergent cohorts from which these data were obtained. As expected, individuals with chronic infections on ART had lower genetic diversity than individuals with chronic infections who were not on ART (log mean entropy = −3.56 vs −3.50, KS test *P* = .02). Identical patterns were observed if participants were split by viral suppression rates (Supplementary Figure 1), reflecting viral suppression rates >95% (1595 of 1662) among treated patients.

### Antiretroviral Treatment Status and Diversity Are Most Important for Predicting Stage of Infection

**Figure 2.**
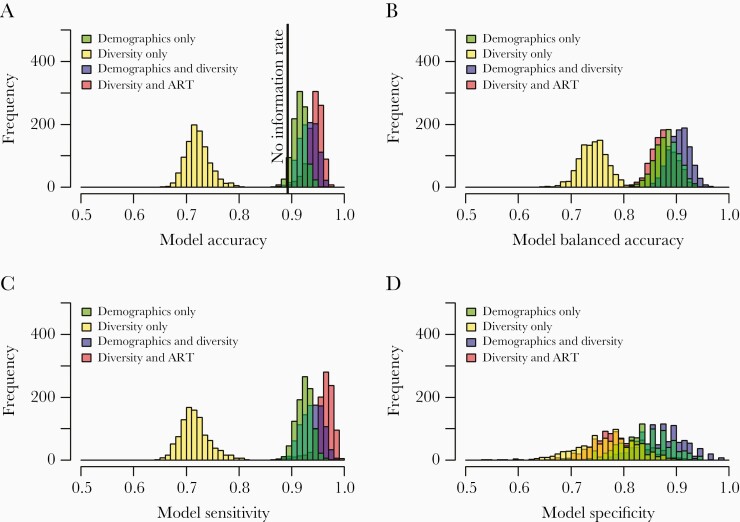
(A) Model accuracy, (B) balanced accuracy, (C) sensitivity, and (D) specificity with cross-validation for 4 models with different sets of predictors (1) demographic/clinical predictors only (age, sex, viral load, and antiretroviral treatment [ART] status), (2) diversity (in each of the 3 genes) only, (3) diversity and demographics, and (4) diversity and ART status. Each model was fitted and evaluated 1000 times, splitting the complete data into training (70%) and test (30%) data each time. The no information rate for accuracy is the proportion of the dominant class (here, 89%). The equivalent no information rate for balanced accuracy would be 50%.

We compared 4 models: (1) a model including a measure of diversity only (for *gag*, *pol*, and *env*), (2) a model including demographic and clinical predictors only (age, sex, ART status, viral load), (3) a model including measures of diversity and ART status, and (4) a model including all available predictors. Diversity calculated using entropy performed slightly better than diversity calculated using π (data not shown), as demonstrated previously [30]; henceforth, we present results only for entropy. In the complete dataset, 89.2% of samples were from chronic infections, meaning that a model predicting all samples to be chronic would have an accuracy of 89.2%. This number represents the “no information rate.” The model based on diversity alone did not predict recency any better than the no information rate, but all 3 other models performed significantly better than the no information rate (Figure 2A). We selected the best model based on balanced accuracy (Figure 2B), which corrects for the difference in size of the 2 classes by maximizing both sensitivity and specificity instead of maximizing the overall rate of correct calls. The model with the highest balanced accuracy included all predictors: log entropy for each of *gag*, *pol*, and *env*, age, sex, log viral load, and ART status as well as interaction terms for diversity and ART status and diversity and viral load, and its specificity was significantly higher than that of the other models (Figure 2D). This latter result indicates than demographic and clinical predictors other than ART were particularly informative for correctly classifying chronic infections. The *gag* region contributed most substantially to the model, followed by *pol*, but inclusion of all 3 regions performed best (data not shown). In more than 1000 cross-validation replicates, the accuracy of the best model was 93.2% (95% confidence interval [CI], 90.0%–96.2%), balanced accuracy was 90.6% (95% CI, 86.7%–94.1%), sensitivity was 93.9% (95% CI, 89.9%–97.6%), and specificity was 87.4%% (95% CI, 78.6%–94.8%). The balanced accuracy of this final model was significantly higher than the balanced accuracy of the next best model, containing only diversity and ART (balanced accuracy = 87.6%; *t* test, *P* < 10^–16^).

### xgboost Can Predict Stage of Infection for Incomplete Cases

**Figure 3.**
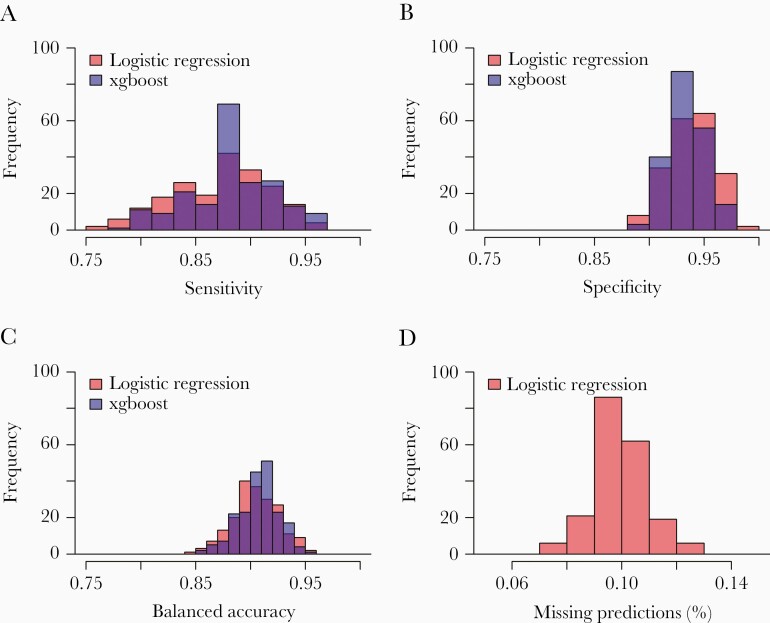
(A) Sensitivity, (B) specificity, (C) balanced accuracy, and (D) percentage of missing predictions for the logistic regression and machine learning models. Statistics are calculated by fitting the model each time to a training dataset, then evaluating it in a test dataset. Note that the xgboost model was always able to predict recency even in the absence of some predictors (D).

Next, we compared the best performing logistic regression model to a machine learning model (xgboost) with the same predictor variables: log entropy for each of *gag*, *pol*, and *env*; and age, sex, log viral load, and ART status. Note that xgboost does not require interaction terms to be detailed explicitly. Models were compared through 200 cross-validation replicates. When optimized for balanced accuracy, the regression and machine learning models performed comparably, with no difference in balanced accuracy, sensitivity slightly higher for the machine learning model, and specificity slightly higher for the regression model (Figure 3A–C). However, demographic and clinical data were not complete for every participant included, and sequence data were not always available for every gene. In instances in which data were missing, the logistic regression model failed to make predictions (Figure 3D). We were able to fit regression model variants, removing 1 predictor (including 1 gene region) at a time, and the model still predicted accurately for those samples that were missing information (data not shown), but such a procedure is time intensive. The xgboost model had good prediction accuracy even for participants with missing data, although missing data is not explicitly imputed.

The sensitivity, specificity, and accuracy statistics in the logistic regression model do not consider cases for which no prediction is made. Our test datasets comprised ~582 cases, and, for a typical model run, the logistic regression model could not predict for approximately 10.01% of cases (Figure 3). xgboost performed well in predicting stage of infection among participants with and without missing data (data not shown).

### Splitting the Data by Treatment Status Improves Recency Prediction

**Figure 4.**
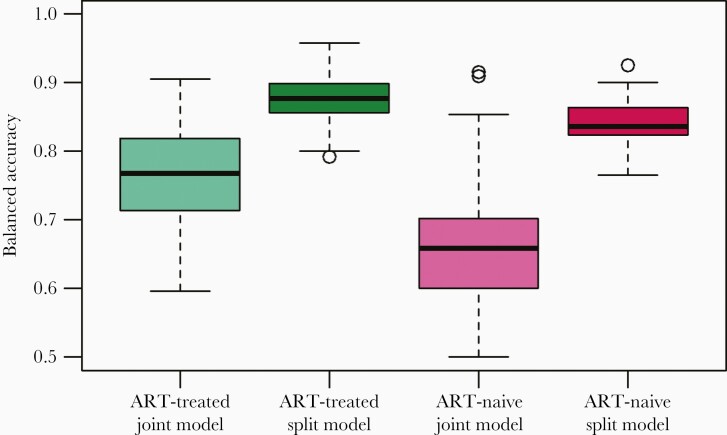
Balanced accuracy of the predicted stage of infection for participants based on antiretroviral treatment (ART) status. In the joint model, the model was fit to all participants regardless of ART status, and ART status was included as a predictor. In the split model, the model was fit separately to ART-treated and ART-naive participants. The split model improved balanced accuracy for both ART-treated and ART-naive participants (*P* < 10–16).

Next, we assessed the sensitivity and specificity of our final model in predicting stage of infection in ART-treated versus ART-naive cases. We examined the distribution of model statistics based on 200 cross-validation tests. Although overall sensitivity and specificity for this model were high, specificity among the ART-naive group was low (34.1%) (Supplementary Figure 2), meaning that the model was not good at identifying ART-naive chronic infections. Similarly, our ability to correctly classify recent infections among ART-treated individuals was subpar (sensitivity = 64.6%) (Supplementary Figure 2). In both cases, numbers within these groups were small as a proportion of total chronic infections (99 of 1735) (Table 1) and of total recent infections (41 of 209), explaining why the model was unable to accurately disentangle that group. Balanced accuracy (the mean of sensitivity and specificity) was significantly improved for both ART-treated and ART-naive individuals by fitting xgboost models and predicting recency status separately on ART-naive and ART-treated individuals (*t* test, *P* < 10–16 for both comparisons) (Figure 4), although sensitivity among ART-naive and specificity among ART-treated were both reduced (all *P* < 10–16) (Supplementary Figure 2). These models separately achieved 91.4% sensitivity and 83.7% specificity among ART-treated individuals and 81.4% sensitivity and 86.9% specificity among ART-naive individuals. Our models performed better in ART-treated participants than ART-naive because our dataset was larger.

### Self-Reported Human Immunodeficiency Virus Testing History in Botswana Is Reliable

Finally, we applied our xgboost model to classify infections diagnosed at the start of BCPP trial. We set out to compare predictions between participants who had documented evidence of a prior negative HIV test within the last year (n = 12), those who reported a negative HIV test within the previous year but had no record (n = 46), and those who reported a negative HIV test more than 1 year prior but who had no record (n = 114). There were twice as many predicted chronic infections among those self-reporting a negative HIV test within the last year with no record (19.6%) than among those who did have a record (8.3%), but the difference was not significant (Fisher test, *P* = .42) (Table 2). The distribution of predicted probabilities of recency for those 2 groups were not significantly different either (KS test, *P* = .97) (Supplementary Figure 3A). In contrast, those who self-reported a negative HIV test more than 1 year ago were significantly more likely to be classified as chronic than those self-reporting a negative HIV test less than 1 year ago (37.7% vs 19.6%, Fisher’s test, *P* = .04) (Table 2), and their recency probability distributions were also significantly different (KS test, *P* = .007) (Supplementary Figure 3B).

**Table 2.** Recency Prediction Among 3 Groups: Those With Evidence of a Negative Test Within the Last Year (n = 12), Those Who Self-Reported a Negative Human Immunodeficiency Virus (HIV) Test Within The Last Year but Had No Record (n = 46), and Those Who Self-Reported a Negative HIV Test More Than 1 Year Ago but Had No Record (n = 114)

Model Prediction	Negative Test <1 Year—With Record	Negative Test <1 Year—No Record	Negative Test >1 Year—No Record
Chronic >1 year	1 (8.3%)	9 (19.6%)	43 (37.7%)
Recent <1 year	11 (91.7%)	37 (80.4%)	71 (62.3%)

## DISCUSSION

We were able to predict the stage of HIV infection within a cohort including participants receiving ART with suppressed viral load. Stage of infection could be inferred from proviral DNA sequence diversity with high accuracy. Our model performed comparably to models using NGS-derived measures of genetic diversity to predict stage of infections among ART-naive participants [13, 15]. Recent infections were identified with a sensitivity of 93.9% and a specificity of 87.4%. Among treated participants, genetic diversity measures (eg, entropy) displayed overlap between recent and chronic infections, but including clinical and demographic data allowed for the groups to be disentangled. A gradient boosting machine learning algorithm provided substantial improvements by classifying stage of infection even among the 10% of participants missing 1 or more predictors.

Estimating time since infection from HIV sequences relies on the steady accumulation of genetic diversity within patients after infection. However, after ART initiation, virus replication is suppressed and sequences from proviral DNA can resemble those present when treatment was initiated [31–33]. As a consequence, classifying infections as recent or chronic when patients are on ART is challenging. Our predictive model achieved a balanced accuracy significantly above 50% regardless of ART status. However, we concede that ART interferes with disease staging, whether using clinical or sequenced-based metrics, and, in agreement, fitting models independently to treated and untreated participants improved predictive ability. Our dataset was skewed, with only a minority of recent infections treated, but such individuals will become more numerous as treatment expands, thus predicting stage of infection among this group is of considerable importance. In fact, future studies may include only treated patients; based on our analyses, staging of infection should still be possible. Additional resolution may require investigation of longitudinal changes in genetic diversity in treated patients, but the cross-sectional data to which our model is fitted reflects the types of data currently available.

The ability to distinguish between recent and chronic infections among participants on ART was in part due to the wealth of demographic and clinical data available from participants in this study; indeed, incorporating this information (and specifically, viral load [34]) has previously been shown to improve prediction of stage of infection based on viral RNA diversity estimates [35]. Inclusion of CD4 count would further improve predictions [36], but CD4 counts were not available for our cohort because HIV treatment is now recommended regardless of CD4 count in Botswana. A substantial proportion of the signal was derived from ART status, but including measures of genetic diversity significantly improved classifications. Consistent with similar analyses [13, 15], we found *gag* and *pol* to be the most informative regions. The *env* region is likely to better resolve time since infection early on, but rapid rates of diversification lead to saturation and loss of signal later in infection [30, 37]. In addition, for highly divergent HIV *env* sequences, alignment remains problematic, impacting estimates of genetic distance. Nonetheless, we concede that although classification accuracy was high in our large dataset, and high enough for population-based downstream applications, it is insufficient for use as a patient-level diagnostic test. Furthermore, the fitted predictive model is heavily dependent on clinical and demographic data, and the ways in which such factors affect disease progression varies across regions [38]. Specifically, our cohorts consisted almost entirely of subtype C infections diagnosed among heterosexuals, and, consequently, our model may not be directly extrapolatable to populations with more rapid transmission, for example, men who have sex with men or injection drug users. We were not able to compare sequencing success rates between recent and chronic infections, nor were we able estimate the sensitivity of the proviral sequencing method, from our sample processing pipeline. Given that the HIV reservoir is smaller among patients put on treatment early [39], potential undersampling of this group could introduce a source of bias into our results.

We applied our algorithm to a subgroup of participants newly diagnosed with HIV at the start of the BCPP trial in Botswana. We found that among those with no HIV test records, those who self-reported a negative HIV test within the previous year were significantly more likely to be classified as recent infections by our algorithm than those who reported a negative HIV test more than 1 year previously. Meanwhile, there was no significant difference in classification between those self-reporting a negative HIV test within the previous year, whether or not they had a record. There was a tendency for patients with a record to be more likely classified as recent, but the difference was not significant. These results, taken together, suggest that self-reported testing history in Botswana is reliable. Studies assessing the accuracy of HIV testing history in sub-Saharan Africa have focused on the reliability of results rather than on timing. Overall, recent studies have similarly found self-reporting of HIV status to be reliable [40, 41]; although an earlier study in Malawi concluded that up to 1 of 3 of HIV-positive individuals may knowingly misreport their HIV status [42]. To our knowledge, ours is the first study that investigates the reliability of self-reporting of timing of HIV tests. In view of the considerable effort put into developing laboratory-based assays for the purpose of recency testing, it is worth emphasizing that self-reporting may also be an increasingly reliable indicator.

## CONCLUSIONS

In conclusion, identifying recent infections (<1 year) using NGS-derived estimates of within-host HIV genetic diversity appears possible even among individuals on ART if additional demographic and clinical data are available. As universal test and treat becomes standard practice, future diversity-based classifiers will increasingly focus on treated populations and will be based on proviral DNA by necessity. These results could enable the detailed examination of the contribution of recent infections to onward transmission in Botswana and other PANGEA sites within the context of the 90-90-90 UNAIDS target.

## Supplementary Data

Supplementary materials are available at The Journal of Infectious Diseases online. Consisting of data provided by the authors to benefit the reader, the posted materials are not copyedited and are the sole responsibility of the authors, so questions or comments should be addressed to the corresponding author.

jiab293_suppl_Supplementary_Materials

## Notes

***Acknowledgments.*** We acknowledge all of the researchers and staff at the Botswana Harvard AIDS Initiative as well as all of the Botswana Combination Prevention Project study participants. We thank 2 anonymous reviewers for their constructive insights.

***Financial support.*** This work was funded in part by the Bill & Melinda Gates Foundation (PANGEA 1: OPP1084362, PANGEA 2: OPP 1175094). This work was also funded by President’s Emergency Plan For AIDS Relief (PEPFAR)/Centers for Disease Control and Prevention (Grant Numbers U01 GH000447 and U2G GH001911; to the Botswana Combination Prevention Project [BCPP] project) and National Institute of Allergy and Infectious Diseases (R01 AI083036; for the Mochudi project). We acknowledge joint Centre funding from the UK Medical Research Council and the Department for International Development (MR/R015600/1).

***Potential conflicts of interest.*** E. V. has an honorary contract with Public Health England (September 2020–present) to conduct work in the Genomic Epidemiology Cell. C. F. reports grants from the Bill & Melinda Gates Foundation during the conduct of the study. Under the grant conditions of the Foundation, a Creative Commons Attribution 4.0 Generic License has already been assigned to the Author Accepted Manuscript version that might arise from this submission. All authors have submitted the ICMJE Form for Disclosure of Potential Conflicts of Interest. Conflicts that the editors consider relevant to the content of the manuscript have been disclosed.

Presented in part: Conference on Retroviruses and Opportunistic Infections March 8–11 2020 (virtual conference) Boston, MA, USA; Dynamics & Evolution of HIV and Other Human Viruses May 10–13, 2020 (virtual conference) San Diego, CA, USA.
