# Supplementary material for: Human Immunodeficiency Virus (HIV) Genetic Diversity Informs Stage of HIV-1 Infection Among Patients Receiving Antiretroviral Therapy in Botswana
Source: J Infect Dis. 2021 Jun 2;225(8):1330–8. doi: 10.1093/infdis/jiab293 (PMC9016439; doi:10.1093/infdis/jiab293)
Supplement: jiab293_suppl_Supplementary_Materials [file jiab293_suppl_supplementary_materials.docx]

HIV genetic diversity informs stage of

HIV-1 infection among patients receiving antiretroviral therapy in Botswana

Supplementary Tables and Figures

Manon Ragonnet-Cronin^1^, Tanya Golubchik^2^, Sikhulile Moyo^3^, Christophe Fraser^2^, Max Essex^3,4^, Vlad Novitsky^3,4,5^, Erik Volz^1^ with the PANGEA Consortium

1. MRC Centre for Global Infectious Diseases Analysis, Imperial College London, London W2 1PG, UK
2. Big Data Institute, University of Oxford, Oxford OX3 7LF, UK
3. Botswana Harvard AIDS Initiative, Gaborone, Botswana
4. Department of Immunology and Infectious Diseases, Harvard T.H. Chan School of Public Health, Boston, MA FXB 402, USA
5. Brown University, Providence RI 02912, USA

Supplementary Table 1: Sequenced gene region count for individuals with known recent and chronic infections.

|  |  | Recent | Chronic |
| --- | --- | --- | --- |
| Total |  | 209 | 1735 |
| Full genome (gag, pol, env) |  | 195 | 1607 |
| gag + pol |  | 4 | 57 |
| gag + env |  | 0 | 9 |
| env + pol |  | 8 | 35 |
| gag only |  | 0 | 16 |
| pol only |  | 0 | 0 |
| env only |  | 2 | 11 |

## Supplementary Figure 1


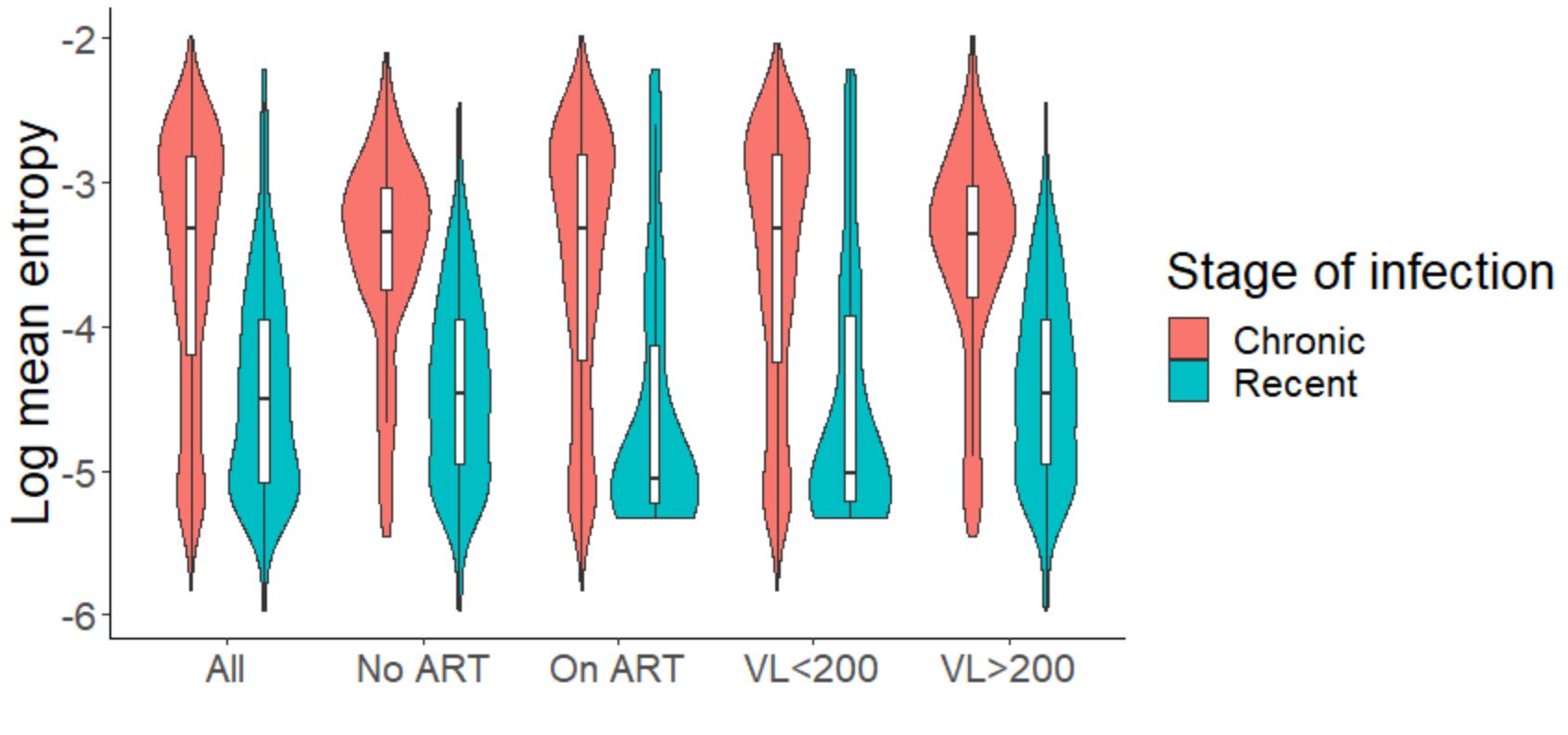


Supplementary Figure 1: Viaplot of log mean entropy for participants based on stage of infection (chronic and recent), ART-status (naïve or treated) and viral loads (suppressed <200, vs unsuppressed >200)

## Supplementary Figure 2


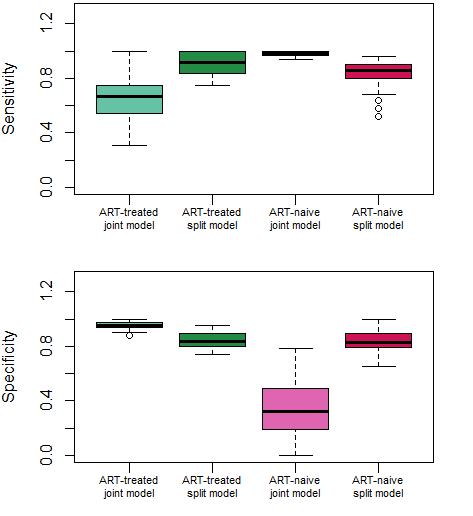


Supplementary Figure 2: Sensitivity and specificity of predicted stage of infection for participants based on ART status. In the joint model, the model was fit to all participants regardless of ART status, and ART status was included as a predictor. In the split model, the model was fit separately to ART-treated and ART-naïve participants. The split model increased sensitivity and decreased specificity for ART-treated participants. The effect was reversed in ART-naïve participants (all p<10-16).

## Supplementary Figure 3


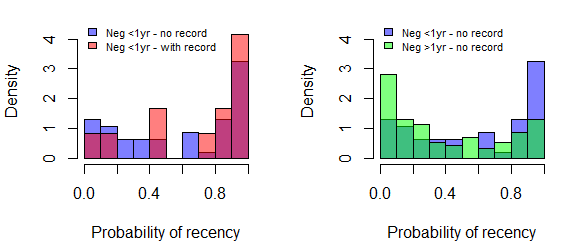


Supplementary Figure 3: Probability distribution of recency prediction among three groups: those with evidence of a negative test within the last year (n=12, in red), those who self-reported a negative HIV test within the last year but had no record (n=46, in blue) and those who self-reported a negative HIV test more than a year ago but had no record (n=114, in green).
